# Reassessing Tuberculosis Treatment Outcomes by Sputum Liquid Culture Testing at the End of Treatment in Karnataka, India

**DOI:** 10.7759/cureus.106165

**Published:** 2026-03-30

**Authors:** Anil S, Satish Ghatage, Kiran K, Shubha Davalgi, Swathi S Aithal, Nirmala A R, Nimra Shireen, Shazia Anjum, Hamsaveni G, Rajeev N Sethumadhavan, Jose J Thomas

**Affiliations:** 1 Department of Health and Family Welfare, State TB cell, Government of Karnataka, Bengaluru, IND; 2 TB Technical Support Network, Government of India - World Health Organization, New Delhi, IND; 3 Department of Community Medicine, JJM Medical College, Davangere, IND

**Keywords:** liquid culture, recurrence, treatment failure, treatment outcome, tuberculosis

## Abstract

Background: Tuberculosis (TB) remains a major global health condition, particularly in low- and middle-income countries. Treatment failure and relapse are of great concern, and treatment monitoring is largely dependent on smear microscopy. Smear microscopy has its limitations, particularly in identifying low bacillary loads and viable bacilli. This study aimed to estimate the prevalence of treatment failure and its associated risk factors among drug-sensitive TB (DSTB) patients using sputum liquid culture testing at the end of treatment.

Methods: A cross-sectional study was conducted among microbiologically confirmed pulmonary DSTB patients between April and June 2024 in six randomly selected districts of Karnataka, India. Sputum samples obtained at the time of completion of treatment were cultured using the Mycobacterium Growth Indicator Tube (MGIT) liquid culture system in three culture and drug susceptibility testing (C-DST) laboratories in the state. Demographic and clinical information were accessed through Ni-kshay, the case-based web-based TB reporting system used in India. Statistical analysis was performed using Jamovi v2.3 (Jamovi Project, Sydney, Australia).

Results: Among 511 patients enrolled, the mean age was 46 ± 17 years, 70.3% were male, 15.3% had a history of TB, 5.5% were HIV reactive, and 20.7% had diabetes. The mean BMI was 18.9 ± 2.8 kg/m². Liquid culture testing found 40 (7.8%; 95% CI: 5.6%-10.5%) patients to be culture positive. Thus, the TB treatment failure rate was 7.8%. Males and patients with a history of TB had relatively increased risks of treatment failure (RR: 1.27 and 1.18, respectively), but these were not statistically significant. Age, BMI, HIV reactivity, and diabetes also did not demonstrate significant associations with treatment failure.

Conclusion: This study highlights the inadequacy of smear microscopy for treatment outcome measurement and shows that end-of-treatment liquid culture significantly enhances the detection of treatment failure. Scaling up the application of high-sensitivity tests, such as liquid culture, at the programmatic level could enhance outcome classification, enable timely clinical interventions, and reduce recurrence and transmission rates. Programmatic investment in sensitive diagnostic technology for diagnosis and treatment monitoring is essential in the strategy toward India’s TB elimination goal.

## Introduction

Tuberculosis (TB) remains one of the leading public health concerns, as it is the top infectious killer in the world [1]. According to the Global TB Report 2024 by the World Health Organization (WHO), an estimated 10.8 million people were affected by TB, and 1.25 million people died from the disease in 2023 [2]. Despite significant progress in controlling TB, the burden remains high in low- and middle-income countries, including India. India, being one of the high-burden countries for TB, contributes around 26% of the global TB burden, with an estimated 2.8 million incident TB patients and 0.31 million deaths from the disease in 2023 [3]. Effective strategies and accurate monitoring mechanisms are essential to eliminate TB by 2030 [4].

TB is a curable disease. Traditionally, programmatic TB treatment outcomes include cure, treatment completed, failure, death, and loss to follow-up [5]. TB cure is defined as a microbiologically confirmed TB patient being smear microscopy-negative at the end of treatment [5]. Around 5-15% of people with TB experience recurrence of disease within one year after being cured. Recurrent TB may be due to reinfection or relapse caused by persistent viable bacilli [6]. Factors such as inadequate treatment regimen, poor adherence to treatment, and emergence of drug resistance can also contribute to the recurrence of TB disease [7]. Thus, accurate assessment methods are crucial for monitoring treatment outcomes and identifying individuals at risk of recurrence [8].

Sputum microscopy is a cost-effective tool for diagnosis and treatment monitoring, particularly in resource-limited settings. The sensitivity of the test is 40-60% compared to solid culture using Lowenstein-Jensen (LJ) medium. Sensitivity can further decrease, especially when the bacterial load is low [9,10]. It is less effective in detecting extrapulmonary TB and cannot differentiate between live and dead bacilli. Sputum microscopy at the end of TB treatment does not rule out immediate recurrence of TB disease, as it fails to detect low-level bacillary presence [10].

Sputum culture, using liquid culture systems such as the MGIT (Mycobacterium Growth Indicator Tube), is another test used to diagnose and monitor TB treatment. Unlike smear microscopy, sputum liquid culture is much more sensitive and can detect bacterial loads as low as 10-100 bacilli/mL [11]. It has a sensitivity of 91% compared to solid culture using LJ medium [12]. A negative sputum culture at the end of TB treatment predicts long-term cure and a lower recurrence rate [13]. If the results are positive, it indicates treatment failure and guides clinicians in modifying the treatment regimen. Despite its high sensitivity, the associated cost and laboratory infrastructure limit its scaling up in resource-limited settings. The National TB Elimination Program (NTEP) in India has currently scaled up the use of liquid culture in the diagnosis and treatment monitoring of people with drug-resistant TB (DRTB) [14].

Although sputum smear microscopy is less sensitive than sputum culture, it is currently used by NTEP to monitor TB treatment among people with drug-sensitive TB (DSTB). In Karnataka, the TB treatment failure rates identified using sputum smear microscopy among all notified DSTB patients in 2021 and 2022 were 0.6% and 0.5%, respectively [3,15]. In contrast, among notified TB patients, 10% and 13% had a history of previous TB treatment in 2022 and 2023, respectively [3,15]. This recurrence of TB disease might be due to the inability to identify treatment failures using less sensitive sputum smear microscopy.

India’s National Strategic Plan for TB elimination is striving to make significant efforts in reducing the TB burden [3]. In this context, sputum culture plays a pivotal role in ensuring accurate treatment outcomes and recurrence-free cure. Therefore, this study aims to estimate the prevalence of treatment failure among patients with drug-susceptible tuberculosis (DSTB), as assessed by sputum culture at the end of treatment, and to evaluate risk factors associated with treatment failure.

## Materials and methods

A cross-sectional study was conducted among pulmonary, microbiologically confirmed DSTB patients completing TB treatment in six randomly selected districts (Yadgiri, Raichur, Hassan, Mandya, Belgaum, and Gadag) of Karnataka. Six districts were selected using a simple random method, based on the culture and drug susceptibility testing (C-DST) laboratory capacity.

Karnataka, a South Indian state, has 32 districts with 2508 functional microscopy centers and 347 nucleic acid amplification testing (NAAT) centers. Upfront molecular testing using NAAT is offered for 35% of presumptive TB patients, while the remaining are diagnosed by microscopy. Sputum smear microscopy is also used for treatment monitoring for DSTB patients at the end of the intensive phase and the continuation phase (end of treatment) of TB treatment. The state has one intermediate reference laboratory (IRL) in Bangalore and two C-DST laboratories in Dharwad and Raichur districts. All three laboratories are quality assured and supervised by the National TB Institute, Bengaluru.

The study included pulmonary, microbiologically confirmed DSTB patients completing TB treatment in six randomly selected districts between April and June 2024. Patients with drug resistance, extrapulmonary TB, or disseminated TB were excluded from the study.

The study population consisted of 511 pulmonary, microbiologically confirmed DSTB patients completing TB treatment. Sputum samples were collected from all eligible participants; hence, a universal sampling method was used. This sample size is adequate to achieve a 95% confidence level and 80% power, assuming a TB treatment failure rate of 1.08%, as reported in a study by Gilmour et al. [16].

TB treatment failure was defined as sputum smear microscopy or culture positivity at five months or later during treatment in patients with drug-sensitive TB [17].

Administrative approval from the State TB Officer, Karnataka, and ethical and institutional review board approval were obtained prior to initiation of the study. Sputum specimens were collected from study participants at the end of treatment after obtaining consent and were transported to the linked C-DST laboratories under a cold chain, as per NTEP guidelines. The sputum specimens were subjected to liquid culture testing using MGIT to monitor treatment outcomes. Demographic details such as age and gender, and other parameters including HIV status, diabetic status, previous history of TB, body mass index, and end-of-treatment culture reports, were obtained from Ni-kshay, the case-based web-based TB reporting system used in India [18]. The confidentiality of subjects was maintained, and patient identifiers were masked during analysis.

Data were analyzed using the open-source analytical software Jamovi v2.3 (jamovi project, Sydney, Australia) [19]. Categorical variables are presented as frequencies and proportions, while continuous variables are presented as mean with standard deviation. Factors associated with TB treatment failure were analyzed using chi-square and Fisher’s exact tests, and relative risk was calculated. A 95% confidence interval for proportions was calculated. A p-value <0.05 was considered statistically significant.

## Results

Out of 511 microbiologically confirmed pulmonary DSTB patients offered end-of-treatment culture, the mean age was 46 ± 17 years, 359 (70.3%) were male, 78 (15.3%) had a previous history of TB, 28 (5.5%) were HIV reactive, 106 (20.7%) were diabetic, and the mean body mass index was 18.9 ± 2.8 kg/m² (Table 1). Of the 511 sputum specimens processed using liquid culture testing with MGIT, 40 (7.8%) were positive. Thus, the TB treatment failure rate by the liquid culture method was 7.8% (95% CI: 5.6%-10.5%). Males had a higher risk of treatment failure than females (RR: 1.27, 95% CI: 0.63-2.53), and previously treated TB patients had a higher risk of treatment failure compared to new TB patients (RR: 1.18, 95% CI: 0.54-2.57). However, these findings were not statistically significant. Age, body mass index, HIV reactivity, and diabetes were also not associated with TB treatment failure (p-value>0.05) (Table 2). 

**Table 1:** Baseline characteristics of microbiologically confirmed pulmonary drug sensitive TB patients completed treatment from April to June 2024 in six districts of Karnataka. *Mean ± standard deviation.

Characteristics	Number (n = 511)	Percentage
Sex		
Male	359	70.3
Female	152	29.7
Previous history of TB		
Yes	78	15.3
No	433	84.7
HIV status		
Reactive	28	5.5
Non-Reactive	483	94.5
Diabetic		
Yes	106	20.7
No	405	79.3
Age (in years)*	46 ± 17	
Body mass index (kg/m^2^)*	18.9 ± 2.8	

**Table 2:** Factors associated with end of TB treatment culture positivity among pulmonary drug sensitive tuberculosis patients in Karnataka from April to June 2024.

Variables	Total	TB treatment failure, N (%)	p-value	Relative risk (95% CI)
Age				
≤40 years	195	16 (8.2)	0.80	1.08 (0.58-1.89)
>40 years	316	24 (7.6)		
Body mass index (kg/m^2^)				
<18.5	250	20 (8.0)	0.88	1.04 (0.57-1.89)
≥18.5	261	20 (7.7)		
Gender				
Male	359	30 (8.4)	0.49	1.27 (0.63-2.53)
Female	152	10 (6.6)		
Previous history of TB				
Yes	78	7 (9.0)	0.68	1.18 (0.54-2.57)
No	433	33 (7.6)		
HIV status				
Reactive	28	1 (3.6)	0.38	0.44 (0.06-3.1)
Non-reactive	483	39 (8.1)		
Diabetic				
Yes	106	5 (4.7)	0.18	0.55 (0.22-1.36)
No	405	35 (8.6)		

## Discussion

TB continues to be a significant global health challenge despite advances in diagnosis and treatment. TB treatment failure and recurrent TB remain challenges in patient management and in reducing TB burden. It is important to use highly sensitive and specific tools for accurate treatment monitoring to determine the correct status of treatment outcome and to achieve the goal of TB elimination in India.

This cross-sectional study aimed to assess the TB treatment failure rate among microbiologically confirmed pulmonary DSTB patients using liquid culture at the end of TB treatment and the risk factors associated with it. Among the study population, the treatment failure rate was 7.8%. The treatment failure rates monitored using sputum smear microscopy among all notified DSTB patients in Karnataka in 2021 and 2022 were 0.6% and 0.5%, respectively [3,15]. However, the treatment failure rates compared here are not of the same cohort. Patients in 2021 and 2022 were tested programmatically by sputum microscopy, whereas the study population was tested using sputum liquid culture. Appropriate identification of treatment failure will help clinicians in further monitoring patients and treating them with the appropriate regimen, which may reduce recurrent TB disease and transmission in the community.

Sputum smear microscopy has a sensitivity of 40-60%, which decreases significantly at lower bacterial loads and is less effective for diagnosing extrapulmonary TB. It cannot distinguish between live and dead bacilli. Sputum culture has better sensitivity, particularly with liquid culture systems such as MGIT, which can detect bacterial loads as low as 10-100 bacilli/ml. Furthermore, negative sputum cultures at the end of treatment have been shown to significantly lower recurrence rates and are highly predictive of long-term cure. A study conducted in Pakistan by Ambreen et al. found that 6% of sputum smear-negative patients had viable Mycobacterium tuberculosis bacilli in sputum identified by culture methods [8]. This suggests that patients believed to be cured after smear microscopy may still have persistent viable bacilli and may be at risk of recurrent TB disease.

In our study, males had 1.27 times higher risk of treatment failure compared to females. A study conducted by Nur NT in Ethiopia found that males had a 4.06 times higher risk, which is much higher than in our study [20]. An Indian study conducted by Prakash Babu et al. also highlighted that males had a higher risk for TB treatment failure [7]. Our study also showed that previously treated TB patients had 1.18 times higher risk of treatment failure compared to new TB patients. A similar finding was seen in a study conducted by Dooley et al. in Morocco [21]. Our study did not find an association of age >40 years, BMI <18.5 kg/m², HIV reactivity, and diabetes with treatment failure. This finding was in contrast to studies by Babu et al., Dooley et al. in Morocco, and Nur in Ethiopia, where older age, malnutrition, HIV reactivity, diabetes, and alcoholism were associated with TB treatment failure [7,20,21]. This may be due to the limited power of the study despite a true association. MacLean et al. also suggested using accurate tests with high sensitivity and specificity for monitoring TB treatment response [22]. NTEP is expanding molecular testing for TB diagnosis; therefore, high-accuracy tests such as liquid culture should also be expanded for monitoring treatment response.

The strength of the study is that it was conducted in a programmatic setting, which provides evidence of replicability. The limitations include its cross-sectional design, inability to follow up clinical treatment failure, and lack of data on treatment adherence and mechanisms of adherence. Additionally, we could not assess failure rates using sputum smear microscopy among the sampled population. Another limitation is that only 40 patients were sputum culture positive, which limited the power of the study to establish associations with risk factors.

## Conclusions

The TB treatment failure rates among pulmonary, microbiologically confirmed DSTB patients observed using the liquid culture method were significantly higher than those observed using sputum smear microscopy programmatically over the years. This evidence emphasizes the need for a sensitive and specific tool for treatment monitoring. As TB control programs are expanding the use of more sensitive molecular testing in diagnosis, similar efforts should be made in treatment monitoring. Treatment monitoring with liquid culture testing for all DSTB patients at the end of treatment would enhance the accuracy of treatment outcomes. Operationally, the capacity of existing C-DST laboratories and sample transportation mechanisms should be considered in universal scale-up. Liquid culture methods will help identify non-responders and treatment failure cases early and manage them appropriately, thereby reducing the risk of TB recurrence and achieving sustained disease control.
